# Feasibility of an Autism-Focused Augmented Reality Smartglasses System for Social Communication and Behavioral Coaching

**DOI:** 10.3389/fped.2017.00145

**Published:** 2017-06-26

**Authors:** Runpeng Liu, Joseph P. Salisbury, Arshya Vahabzadeh, Ned T. Sahin

**Affiliations:** ^1^Brain Power, Cambridge, MA, United States; ^2^Department of Electrical Engineering and Computer Science, Massachusetts Institute of Technology, Cambridge, MA, United States; ^3^Department of Psychiatry, Harvard Medical School, Boston, MA, United States; ^4^Department of Psychology, Harvard University, Cambridge, MA, United States

**Keywords:** augmented reality, virtual reality, feasibility, tolerability, smartglasses, autism spectrum disorder, education, stimulant

## Abstract

**Background:**

Autism spectrum disorder (ASD) is a childhood-onset neurodevelopmental disorder with a rapidly rising prevalence, currently affecting 1 in 68 children, and over 3.5 million people in the United States. Current ASD interventions are primarily based on in-person behavioral therapies that are both costly and difficult to access. These interventions aim to address some of the fundamental deficits that clinically characterize ASD, including deficits in social communication, and the presence of stereotypies, and other autism-related behaviors. Current diagnostic and therapeutic approaches seldom rely on quantitative data measures of symptomatology, severity, or condition trajectory.

**Methods:**

Given the current situation, we report on the Brain Power System (BPS), a digital behavioral aid with quantitative data gathering and reporting features. The BPS includes customized smartglasses, providing targeted personalized coaching experiences through a family of gamified augmented-reality applications utilizing artificial intelligence. These applications provide children and adults with coaching for emotion recognition, face directed gaze, eye contact, and behavioral self-regulation. This preliminary case report, part of a larger set of upcoming research reports, explores the feasibility of the BPS to provide coaching in two boys with clinically diagnosed ASD, aged 8 and 9 years.

**Results:**

The coaching intervention was found to be well tolerated and rated as being both engaging and fun. Both males could easily use the system, and no technical problems were noted. During the intervention, caregivers reported improved non-verbal communication, eye contact, and social engagement during the intervention. Both boys demonstrated decreased symptoms of ASD, as measured by the aberrant behavior checklist at 24-h post-intervention. Specifically, both cases demonstrated improvements in irritability, lethargy, stereotypy, hyperactivity/non-compliance, and inappropriate speech.

**Conclusion:**

Smartglasses using augmented reality may have an important future role in helping address the therapeutic needs of children with ASD. Quantitative data gathering from such sensor-rich systems may allow for digital phenotyping and the refinement of social communication constructs of the research domain criteria. This report provides evidence for the feasibility, usability, and tolerability of one such specialized smartglasses system.

## Background

Autism spectrum disorder (ASD) is a childhood-onset neurodevelopmental disorder with a rapidly increasing prevalence. ASD is now thought to affect as many as 1 in 68 children, and over 3.5 million people, in the United States (1). The economic impact of ASD is substantial with an aggregated total cost in the United States of approximately $236 billion (2). This figure includes direct and indirect costs from a variety of sources, including medical care, special education, and lost parental productivity (2, 3). Many children with ASD benefit from intensive behavioral interventions, although the costs are often prohibitive, ranging between an additional $40,000 and $60,000 per child per year in the United States (4).

The rapid rise in the number of individuals diagnosed with ASD has strained the limited behavioral therapy resources, and many children face considerable delays in obtaining these much-needed interventions (5). These interventions are crucial given that there are no medical treatments to improve the deficits seen in ASD. ASD is principally characterized by impairments in social communication, and the presence of repetitive and restrictive behaviors (6). Evidence-based behavioral therapies have sought to address these deficits by focusing on improving communication, social interactions, and reducing challenging behaviors (7). Vital socially salient information is transmitted through facial emotions, expressions, eye gaze, and other nuanced social cues. Unfortunately, many people with ASD have been found to demonstrate deficits in facial processing, potentially accounting for some of their difficulties in social communication (8).

The high demand for therapeutic autism services combined with recent technological advances have led to a rapid increase in attempts to deliver such interventions through digital modalities (9). Compared to traditional behavioral therapies, evidence for these digital interventions are limited, but there are some research studies to suggest that digital interventions may improve specific skills and behaviors in people with ASD (Table 1).

**Table 1:** Evidence for digital interventions in people with autism spectrum disorder.

Skills and behaviors aided by digital interventions	
Social and emotional skills	(10–13)
Face recognition skills	(14)
Adaptive behaviors	(10–12, 15)
Vocational behaviors	(16)
Academic skills	(17)
Communication skills	(11)
Challenging behaviors	(10)

Furthermore, children with ASD may be especially receptive to such digital interventions (17, 18). Children with ASD have been shown to have a preference for electronic media (19), game-like elements (20), computer-generated speech (21), and those with particular visual strengths may be especially adept at engaging with digital modalities (22). Current diagnostic and therapeutic approaches seldom rely on quantitative data measures of symptomatology, severity, or condition trajectory, and it is hoped that digital tools may help to provide such data. Emerging technology and mobile sensor-rich devices may help to deepen and refine new paradigms for understanding social communication, such as those outlined within the social processes construct of the National Institute of Mental Health’s Research Domain Criteria (RDoC) (23) and to undertake digital phenotyping and subtyping of behavioral conditions (24).

Some of the most promising technologies include augmented reality, and some reports have highlighted the early use of augmented reality interventions in children with ASD (25–27). The Brain Power System (BPS) is a smartglasses-based augmented reality system for children and adults with ASD to teach themselves life skills that may facilitate or enhance self-sufficiency. The BPS consists of a number of gamified applications; the social communication module includes applications designed to teach users to recognize facial emotional expressions and to attend to the faces of others. The BPS collects quantitative data about the user’s environment and interactions through the use of an array of inbuilt sensors and analyzes these data through the use of artificial intelligence (AI), including the use of Affdex emotion AI (Affectiva, Boston, MA, USA) (28). This report will explore the feasibility and usability of the BPS through caregiver interview, staff observation, and structured validation with autism rating scales.

The BPS was developed in response to several areas of need highlighted by people with ASD and their families. One of the most frequently encountered concerns by parents was a feeling of “disconnection” from their children during conversations. This sense of disconnection is likely related to several previously outlined social communication deficits in ASD, including deficits in social-emotional reciprocity, non-verbal communicative behaviors used for social interaction, and deficits in developing, maintaining, and understanding relationships (6).

## Methods

In this report, the feasibility of the BPS was assessed when it was used to provide a single session of a behavioral coaching intervention to two males with ASD. The tolerability, usability, and behavioral changes associated with the intervention were assessed *via* a validated autism inventory, and subjective caregiver and user report. The aberrant behavior checklist (ABC) was the validated autism inventory used in this study (29).

### Users

The BPS system was trialed on two male users aged 8 and 9 years of age who had a specialist-derived clinical diagnosis of ASD according to the DSM-5 criteria (6). Both users screened positive for ASD when assessed with the Social Communication Questionnaire (30), a validated caregiver questionnaire of ASD symptoms that is frequently used as a screener for entrance to research studies (31). User demographics are laid out in Table 2. Each participant was accompanied by their caregiver to the intervention session.

**Table 2:** Demographics of users.

	User A	User B
Age	8 years 7 months	9 years 9 months
Gender	Male	Male
Diagnosis	Autism spectrum disorder (ASD)	ASD
Age of diagnosis	7 years 9 months	6 years 8 months
Prior smartglasses experience	None	None
Corrective eye-wear	None	None
Schooling	Mainstream public school with special academic supports	Mainstream public school with special academic supports
Prior intervention(s)	Occupational therapy, speech and language therapy, social skills training, cognitive behavioral therapy, psychotherapy, dietary modification, anxiolytic medication	Psychotherapy

### Brain Power System

**Figure 1:**
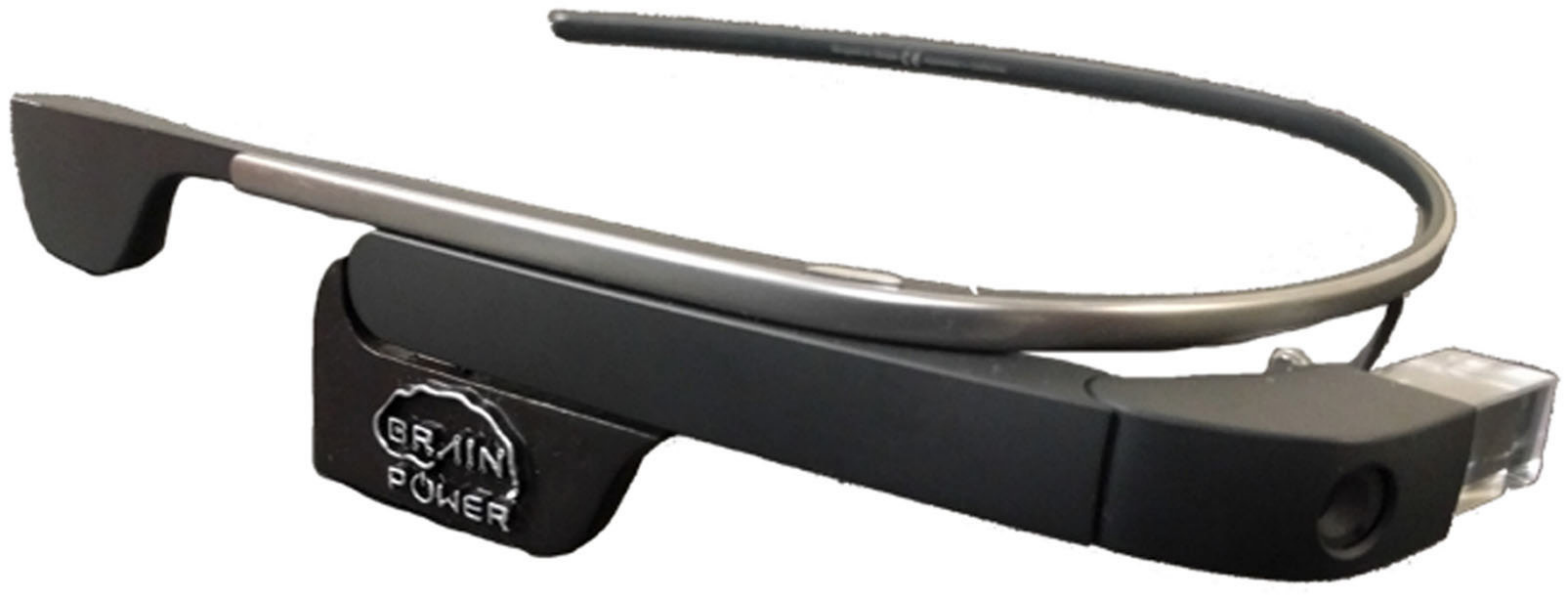
Brain Power System (partial side view).

The BPS is a smartglasses-based behavioral aid designed to help children and adults with ASD with emotional understanding, face directed-gaze, eye contact, and self-control. The BPS is a combination of hardware and software add-ons that may be integrated onto a variety of smartglasses platforms. The physical attributes of the BPS in this report are shown in Figure 1. The BPS includes a family of gamified applications and has onboard sensors that it uses to capture real-time data across several different categories, such as movement, physiology, in-app performance, video, and audio. Software analysis of data from the smartglasses’ inbuilt gyroscope and accelerometer allows for calculation and real-time tracking of a user’s head direction and movements. The BPS can also give real-time visual and auditory feedback to users *via* a small computer screen above the right eye and a bone conduction speaker behind the right ear.

Brain Power System applications have been designed with the goal of enhancing social and cognitive skills development in children and adults with ASD. Face Game and Emotion Game are two gamified BPS applications and are designed to help children improve their ability to engage in face and eye directed gaze and to recognize facial emotion, respectively.

#### Face Game and Emotion Game Apps

Faces are a rich source of socially salient information and humans are drawn to attend to faces, and to gaze at facial features in a stereotypical manner (32). People with ASD have been found to have reduced attention to the faces of others, and compared to their typically developing peers, may demonstrate altered facial feature gaze patterns (33, 34). Two key theories have been put forward to help explain the diminished eye contact in autism, gaze aversion, and gaze indifference. Gaze aversion suggests that the individual with ASD actively avoids eye contact as it is threatening, anxiety-inducing, or linked to physiologic overstimulation (35–37). Gaze indifference, on the other hand, supports the notion that reduced eye contact in ASD may be a passive phenomenon, where the eyes of others are not seen as being engaging or a relevant stimulus (38). Certainly, given the heterogeneity seen in ASD, different theories may hold true for different individuals.

*Face Game* aims to coach users to attend visually to another person’s face while attempting to overcome the suspected challenges identified by both gaze indifference and gaze aversion. *Face Game* uses gamified augmented reality to increase user engagement and interest in human faces, addressing an underlying challenge in gaze indifference. *Face Game* is also designed to help address some of the proposed challenges described in gaze aversion theory. *Face Game* is designed to allow users to have a comfortable starting point, using videogame engagement principles, but proceeding to gradually help users to engage and interact with human faces in the real-world. *Face Game* provides for multiple different levels, and as the user progresses, the digital elements gradually become more subtle, while real-world interactions are emphasized.

*Face Game* applies computer vision algorithms to a real-time camera feed and detects human faces anywhere in the user’s field of view. These detected human faces are then overlaid with an augmented reality cartoon face in a manner that attempts to engage the user and attract his or her attention. When the user turns to look directly at the augmented reality cartoon, it gradually fades, revealing the underlying human face. At this time, *Face Game* rewards the user with “points.” The points rapidly tally for a period while the user’s gaze is directed at their partner’s face. Point accumulation eventually slows and then stops increasing to prevent rewarding and, therefore, promoting staring. The user can then repeat the process of looking away and looking back at the face in order to accumulate more points. The central region of the face—for instance, the eye regions—contains the most socially salient information. *Face Game* provides a higher point reward when users look closer to the more socially salient central region of the face. Closed loop feedback is possible through the collection of in-app metrics and quantitative sensor-data, allowing for customization of game difficulty and rewards.

During typical early childhood development, infants rapidly become able to recognize expressed facial emotions. Facial emotions carry with them important social information about a person’s affective state, intentions, and surroundings (39). New technology may be particularly useful in assessing and measuring facial expressions in a manner that may improve the social communication constructs of RDoCs (23).

Facial emotion recognition appears to be impaired in ASD (40) and reduced emotional understanding is linked to reduced real-world social behavior and adaptive functioning (41). Emotion recognition training may be effective in ASD, however, generalizability to real-world situations is unclear (40). *Emotion Game* was developed as a means of helping users assess and identify different human facial emotions. The aim of *Emotion Game* is to help augment emotion recognition training provided by therapists. Like *Face Game, Emotion Game* also detects human faces, and through software assessment, can accurately identify a range of human facial emotional expressions. *Emotion Game* uses a visual display to prompt the user to correctly identify the facial emotion in the detected human face by presenting them with two emoticon choices. Users are instructed to choose the correct emoticon with a slight tilt of their head. The BPS detects the head movement and awards points depending on the result of the selection. The experience can also feature deeper gamified elements, such as customized on-screen rewards, and adapts to individual performance based on data-driven closed-loop feedback.

### Experimental Design

Two boys with ASD had a single BPS coaching session with two BPS applications, *Face Game* and *Emotion Game*. This report is part of a larger ongoing IRB-approved clinical trial program.

The coaching session consisted of an initial orientation period where the users and caregivers became familiar with the system had the opportunity to wear the device. Once they were comfortable with the device, the user and caregiver had discrete periods of time to learn to play *Face Game* and *Emotion Game*. The participants, caregivers, and study staff had the opportunity to pause or stop the coaching session at any time for any reason including bathroom breaks, food or water breaks, changes in behavior, and lack of tolerability of device. During the intervention, there was additional external audio and video monitoring in place.

Outcome measures consisted of the ABC and a caregiver and user post-intervention interview. The ABC is an empirically developed scale designed to measure behavioral symptoms in individuals with developmental disorders across five domains: irritability/agitation; lethargy/social withdrawal; stereotypic behavior; hyperactivity/non-compliance; and inappropriate speech (29). The ABC was conducted pre-intervention to obtain a baseline measure, and also at 24 h post-intervention. The post-intervention ABC assessed the entire 24 h immediately following intervention. The total ABC score and subscale scores were calculated. Immediately following the intervention, every caregiver had a post-intervention interview. The caregiver interview assessed how the user had interacted with the BPS, exploring feasibility, tolerability, and usability. The caregivers were also asked to identify changes in the user’s social communication and behavior, including questions about perceived changes in emotional connection, behavioral issues, and verbal and non-verbal communication.

### Ethical Considerations

This report was conducted as part of the “Brain Empowerment—Youth Oriented Unconstrained Reality for Social Engagement and Lifeskills Formation Study” approved by Asentral, Inc., Institutional Review Board (Newburyport, MA, USA). The children’s participation in this study was discussed with their legal guardian and informed consent was obtained. The guardians were informed that they could withdraw consent at any time and for any reason.

## Results

Both of the users and their respective caregivers completed the intervention session without any adverse effects noted by the study staff or reported by the participant and caregiver.

### Caregiver Report

Caregivers had a post-intervention structured interview to assess the feasibility and functionality of the BPS. The BPS was felt to have both high tolerability and engagement. Caregivers also felt that both males found the system enjoyable and fun to use (Table 3). The caregivers were also interviewed about their perception of changes in the user’s communication, interaction, and behavioral control during the intervention (Table 4). Additionally, caregivers were asked to rate their own stress level. Both caregivers reported users had improved non-verbal communication, eye contact, and social engagement. Both caregivers felt that verbal communication was unaffected. One caregiver reported decreased emotional connection and behavioral control, while the other noted improvements in both of these areas. One caregiver felt their own stress levels were greatly improved.

**Table 3:** Caregiver report on user interaction with Brain Power System.

	User A	User B
Level of engagement with device	Very high	Very high
Level of tolerability of device and apps	Very high	High
Level of enjoyment	Very high	Very high
Ease of use	Very high	High
Level of interaction with device	Very high	High

**Table 4:** Caregiver perceptions of user and caregiver emotional and behavioral change.

	User A	User B
Non-verbal communication	Greatly improved	Improved
Verbal communication	Unchanged from baseline	Unchanged from baseline
Emotional connection	Greatly improved	Diminished
Eye contact	Greatly improved	Improved
Behavioral control	Improved	Greatly diminished
Social engagement	Greatly improved	Improved
Caregiver stress levels	Greatly improved	Unchanged from baseline

### Aberrant Behavior Checklist

**Figure 2:**
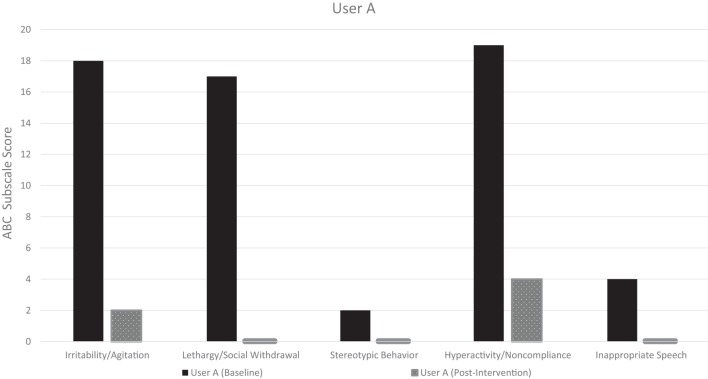
User A: pre- and post-intervention aberrant behavior checklist (ABC) subscale scores.

**Figure 3:**
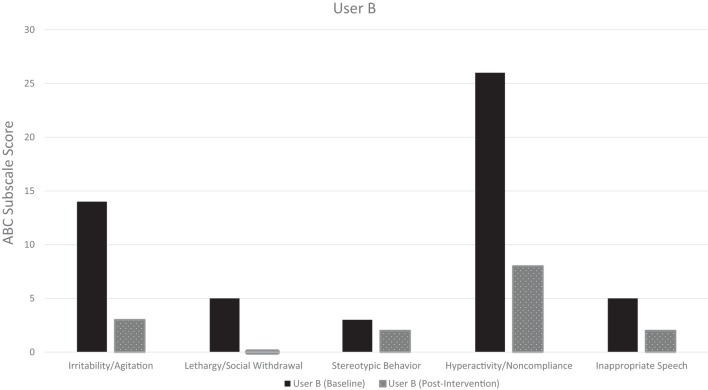
User B: pre- and post-intervention aberrant behavior checklist (ABC) subscale scores.

The pre-intervention ABCs for both User A and User B demonstrate considerable symptom burden across all subscales, with a total baseline symptom score of 60 and 53, respectively (maximum score 174) (Table 5). The post-intervention ABC, which covered the 24 h post-intervention, demonstrated improved symptoms across all subscales in both users. Additionally, both users had a zero post-intervention ABC score in the lethargy/social withdrawal subscale, and User A had a zero score in the inappropriate speech and stereotypic behavior subscales. Both users were noted to have had a substantial decrease in the hyperactivity/non-compliance subscale, the largest subscale contributor to their baseline ABC total score. The pre-intervention and post-intervention ABC-subscale scores for User A and User B are visualized in Figures 2 and 3, respectively.

**Table 5:** Aberrant behavior checklist (ABC)-subscale score pre- and post-intervention.

ABC-subscale	User A		User B	
	Pre-intervention (baseline)	24 h post-intervention	Pre-intervention (baseline)	24 h post-intervention
Irritability/agitation (max. 45 points)	18	2	14	3
Lethargy/social withdrawal (max. 48 points)	17	0	5	0
Stereotypic behavior (max. 21 points)	2	0	3	2
Hyperactivity/non-compliance (max. 48 points)	19	4	26	8
Inappropriate speech (max. 12 points)	4	0	5	2

## Discussion

Augmented reality interventions provide an opportunity to enhance social communication in ASD, and our report demonstrates preliminary evidence of usability and feasibility of one combination of smartglasses and augmented reality technology. Both users managed to use the device without any observable adverse effects, and the BPS was found to be well tolerated, easy to use, and highly engaging according to the caregivers. Caregivers also noted that the users had improved social interactions, through improvements in non-verbal communication, social engagement, and eye contact while using the BPS. It was reassuring to see that ABC scores 24 h post-intervention did not get worse, when compared to the pre-intervention baseline. In fact, it was noted that both children had considerable reductions in symptoms across all five ABC subscales, including irritability/agitation, lethargy/social withdrawal, stereotypic behavior, hyperactivity/non-compliance, and inappropriate speech. While these findings highlight promising feasibility, and usability, and may also suggest some improvement to subjective and objective behavior, there are a number of notable limitations. First, the intervention was tested in two young males, and our findings cannot be expected to generalize to the broader ASD population, which is highly demographically and clinically heterogeneous. Additionally, in this report, the primary aim was to assess feasibility through the use of a single intervention session. To further understand the improvements in ASD symptoms as measured by the ABC, larger studies will be required. Our ongoing research efforts are investigating the outcomes of repeated coaching sessions, in addition to longitudinal monitoring of the ABC to see if there are sustained changes to the ABC based on the use of these interventions. We will also need explore how females with ASD may respond to the BPS, as sex and gender may influence symptoms presentations, and we should be mindful of the range of sexually dimorphic differences in social communication, memory, and cognitive flexibility (42).

When using technologies like the BPS, a vast amount of user data is collected through the inbuilt smartglasses sensors, and analyzed through the applications. These quantitative datasets, encompassing visual, audio, physiologic, and movement data around social interactions, may allow for deeper insights into baseline social communication deficits, and improvements in interventions. Through the use of machine learning we may be able to adopt a data-driven approach to identifying optimal therapeutic and educational approaches for each individual user.

Smartglasses may offer a number of distinct advantages compared to applications delivered *via* smartphones. With smartglasses, users are heads-up as opposed to immersed in a screen, and they remain hands-free, thus able to use their hands to engage in both non-verbal social communication and undertake educational/occupational tasks. This type of mobile and lightweight technology allows users to coach themselves in the privacy of their own home, and whenever is most convenient. Finally, such technology can be rapidly scaled to meet demands.

## Conclusion

The ASD community has considerable difficulty accessing effective and timely therapeutic interventions. This situation may potentially be addressed through the use of digital interventions, such as augmented reality. Augmented reality may be especially effective as it may deliver visual and auditory cues while the user is simultaneously engaged in natural or structured social interactions. Additionally, wearable technologies contain sensors that can be used to record and quantitatively monitor a user’s interaction. This report highlights the need for further research into the use of augmented reality technologies as a therapeutic tool for people with ASD. To the authors’ knowledge, this is the first published report of the use of augmented reality smartglasses, such as Google Glass, as a behavioral aid in a pediatric population. In the two reported cases, augmented reality smartglasses demonstrated high feasibility, usability, and tolerability. Overall, the results are encouraging but should be considered in the context of the limitations outlined.

## Ethics Statement

This report includes data from human participants who used the Brain Power System. The use of the Brain Power System by children and adults with autism is under AIRB 2015-405A; “Brain Empowerment – Youth Oriented Unconstrained Reality for Social Engagement and Lifeskills Formation,” IRB-approved by Asentral INC IRB, who is affiliated with the Commonwealth of Massachusetts’ Department of Public Health.

## Author Contributions

NS is the inventor of the Brain Power System. JS, AV, and NS helped to design and create the intervention. AV was the lead on the writing of this technology report, with additional review and contributions by JS, RL, and NS.

## Conflict of Interest Statement

This report was supported by Brain Power, a neurotechnology company developing a range of augmented reality technologies for smartglasses.
